# Making allyship visible: evaluation of a faculty development DEI curriculum

**DOI:** 10.1080/10872981.2023.2241182

**Published:** 2023-07-30

**Authors:** Jessica Bunin, Jonathan M. Scott, Ryan Landoll, Jessica T Servey, Abigail Konopasky

**Affiliations:** a Dean’s Office, Uniformed Services University of the Health Sciences, Bethesda, MD, USA; b Department of Medical Education, Geisel School of Medicine at Dartmouth, Hanover, NH, USA

**Keywords:** Diversity, equity, inclusion, justice (DEIJ), curriculum development, allyship, faculty development

## Abstract

Undergraduate medical learners from historically marginalized groups face significant barriers, which was made concrete at our institution when a student presented her research indicating that Black students felt unsure about which faculty members to approach. To better support our students, we used Kern’s model for curriculum development and a critical pedagogy approach to create a Faculty Allyship Curriculum (FAC). A total of 790 individuals attended 90 workshops across 16 months and 20 individuals have completed the FAC. A majority of participants reported they felt at least moderately confident in their ability to teach learners who are underrepresented in medicine, mentor learners who are different than they are, and teach allyship topics. An informal content analysis of open-ended responses indicated changes in awareness, attitude, insight, and use of language and being more likely to display advocacy. For others considering creating a similar program, partnering with an existing program allows for rapid implementation and reach to a wide audience. We also recommend: beginning with a coalition of willing learners to quickly build community and culture change; ensuring that the curriculum supports ongoing personal commitment and change for the learners; and supporting facilitators in modeling imperfection and upstanding, ‘calling in’ rather than ‘calling out’ learners.

Making Allyship Visible: Evaluation of a Faculty Development DEI Curriculum Jessica Bunin, Jonathan Scott, Ryan Landoll, Jessica Servey, Abigail Konopasky

## Introduction

Undergraduate medical learners from historically marginalized groups like Black, Indigenous, and People of Color (BIPOC); Lesbian, Gay, Bisexual, Transgender, Queer or Questioning, Intersexual, Asexual and other sexual and gender minorities (LGBTQIA+); persons with disabilities; and women face more barriers than white, heterosexual, cisgender, or male students [1–7]. A student at our institution, an undergraduate medical school with faculty at geographically dispersed educational sites across the nation, presented her research findings to our diversity committee. They revealed that Black students at our institution felt isolated and were unsure of which faculty members were safe to approach, particularly in the context of violence against Black bodies and the social movements that emerged in 2020 (see original study for more details [8]). As a result of this, an intervention team consisting of the curriculum subcommittee of our school of medicine diversity committee and our faculty development department saw the need to create a *visible* group of faculty who are ‘safe’ for historically marginalized learners at our institution. Below, we outline our approach to this problem, our intervention outcomes, and lessons learned.

## Approach

Using Kern’s model for curriculum development [9], the intervention team created a Faculty Allyship Curriculum (FAC) to better support our learners (see Table 1 and supplementary material 1). To complete the FAC, participants were required to attend 9 hours of relevant faculty development, attend a diversity journal club session and write a related reflection, and write a final reflection. A nine hour commitment (6 × 90 minute workshops) was chosen to ensure longitudinal participation of faculty while ensuring the program remained achievable for busy clinicians. Completion of faculty development workshops was tracked in the University’s Faculty Development Self Service Site by program administrators. Upon completion, they received a certificate and lapel pin. Faculty participation in the FAC was voluntary. We took a critical pedagogy approach [10] to this curriculum development process, promoting authentic dialogue, valuing all participants, sharing stories, creating cognitive disequilibrium, and openly discussing and analyzing our own privilege and biases. This allowed us to develop and facilitate materials through the lens of social justice. Full procedures for running the FAC are available in supplemental materials 1. Our institution’s IRB determined this study ‘not research’ and waived consent.

**Table 1.** Application of Kern’s model of curricular development to our context.

Problem	Historically marginalized student populations feel isolated and unable to identify safe allies and mentors
Targeted Needs Assessment	Student research project and conversations with faculty and student stakeholders
Goals and Objectives	Goal: create a cohort of faculty trained to support historically marginalized learners and visually signal their presence Objectives for faculty: Knowledge • Explain common key terms associated with literature on diversity, equity, and inclusion • Summarize the impacts of institutional racism, prejudice, and discrimination on academic medicine Skills • Analyze their own identities and the associated impact of intersectionality and privilege in shaping their individual experiences • Show their commitment to promoting an inclusive workplace and educational environment for students Attitudes • Demonstrate the ability to have empathetic and supportive conversation with students from diverse backgrounds • Illustrate how their own teaching and scholarship will support allyship at our school
Educational Strategies	Virtual and live 90 minute workshops with lecture, reflection, video-based discussion, case-based learning, critique of role-play and small group discussion elements (9 hours of allyship faculty development required) Virtual Diversity, Equity and Inclusion (DEI) Journal Club followed by written reflection Summary reflection of lessons learned and commitment to action
Implementation	Curriculum mapping of goals and objectives to existing faculty development curriculum and creation of new curriculum for unmet goals. Content creator of each workshop conducted a 4-hour training session for facilitators, with opportunities for facilitators to reflect and practice Create calendar of Faculty Development offerings related to Allyship Use existing Faculty Development Self Service Site to track participation in workshops Marketing in established newsletter and faculty development road shows Create calendar of Virtual DEI Journal Clubs Create repository for tracking of submitted reflections Track completion of FAC through collaboration of administrators Creation and approval of FAC completion certificates and lapel pins
Evaluation	Formal written evaluation of each Allyship Workshop Formal written program evaluation after 16 months of FAC participation

## Intervention outcomes

A total of 90 FAC workshops covering 11 topics were offered over the 16 months of this evaluation. Forty of those workshops were offered virtually during the first week of each month to accommodate a geographically dispersed National Faculty. A total of 790 unique individuals attended at least one workshop, live or virtually. To date, 20 individuals have completed the FAC. Formative assessments, including written evaluations, were distributed immediately following each workshop and a summative program evaluation was completed 16 months after the first faculty development workshop (see supplemental materials 2 and 3 for workshop and evaluation questionnaires).

Of the 40 workshops offered virtually, there were 773 learners (not unique learners; learners may have attended more than one workshop). There was a 39% (301/773) response rate on the individual workshop evaluations. Of these 99% (299/301) of respondents reported that workshops were excellent or good.

**Figure 1.**
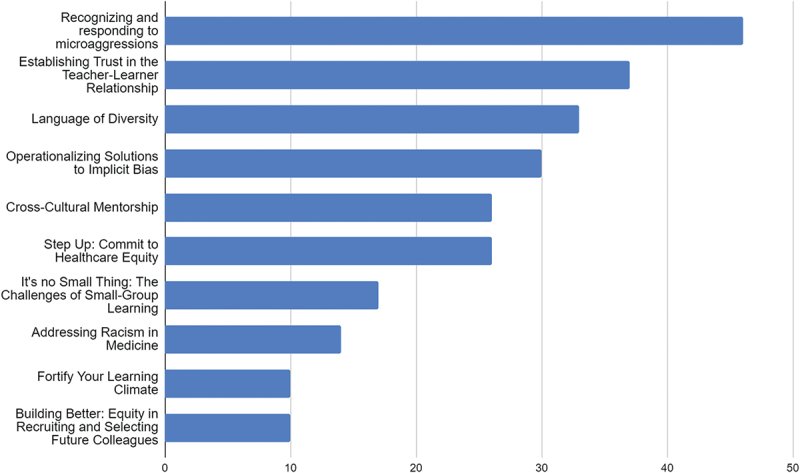
Number of Participants for Each Workshop.

For the overall program evaluation, there was a 10% (75/790) response rate. Figure 1 illustrates the workshops survey respondents took. Most respondents (91%) reported that FAC impacted their teaching practice somewhat or to a great extent. A majority of participants reported they felt at least moderately confident in their ability to teach learners who are underrepresented in medicine (77%), mentor learners who are different than they are (84%), and teach allyship topics (71%).

We conducted an informal inductive content analysis of responses to open-ended questions, guided by Elo and Kyngas [11]. When asked what they were most proud of since beginning the Allyship program, respondents discussed (1) changes in their awareness, attitude, insight and use of language; (2) using allyship materials in teaching, curricular development, or scholarly activity; (3) using what they learned in mentorship, supervision, and leadership; and (4) being more likely to engage in advocacy and display ‘upstander’ behaviors [12] in response to microaggressions. In response to the question about what was most helpful about the program, respondents mentioned increased ability to take others’ perspectives; the presentation of practical tools and action plans for making change; and exposure to the language of diversity.

## Lessons learned

The Allyship curriculum continues to be a driving force for social justice at our institution; faculty regularly comment to us what a difference these spaces to discuss and enact equity make in their practice as teachers, colleagues, and clinicians. For others considering creating a similar program, beginning with a coalition of willing learners (versus mandatory training), as we did, quickly builds community and culture change across the institution. However, participants have noted informally and through the evaluation that our next step must be creating materials for more resistant faculty members. Another lesson learned includes the importance of starting with the voices of historically marginalized students – beginning with learners’ lived experiences grounded the design in their needs and guided our ongoing curriculum development and evaluation. We also strongly recommend partnering with a robust and well-respected program that already exists (for us, faculty development) to reach a wide audience, ease the administrative burden, and promote rapid implementation. Finally, we have recognized the importance of ensuring the curriculum is focused on facilitating ongoing personal commitment and change. To avoid concerns about the curriculum and its associated visible recognition program as performative, the requirements to participate in a journal club and self-reflection in addition to longitudinal course attendance helped to promote authentic learning.

Critical to our intervention were the workshop facilitators, who we trained to ensure psychological safety both for learners from marginalized groups and for all levels of FAC learners. We struck this difficult balance by modeling imperfection and upstanding as facilitators, letting learners know that we were aiming for progress rather than perfection. This accommodating ‘calling in’ versus non-accommodating ‘calling out’ is a cornerstone of our approach [13]. Like others, we found this work carried a risk of burnout [14,15], so we intentionally built community among facilitators, offering them support and urging them to support each other.

A limitation to the evaluation of this curriculum was the 10% response rate on the 16-month program evaluation. This was due in part to the frequent turnover of faculty at our institution (most faculty in the military transition to different institutions every 3 years). As such, we are unsure that our survey reached all participants. Additionally, only one reminder was sent for survey completion. In the future, we would send out more reminders.

As we move forward with our curriculum, we are updating and adding workshop topics, broadening our facilitator pool using a train-the-trainer model, improving our assessment methods, and broadening advertisement of the program.

## Supplementary Material

Supplemental Material

## References

[1] Menon A. Sexism and sexual harassment in medicine: unraveling the web. J Gen Intern Med. 2020;35(4):1302–4. doi: 10.1007/s11606-019-05589-0 31832928 PMC7174475

[2] Dimant OE, Cook TE, Greene RE, et al. Experiences of transgender and gender nonbinary medical students and physicians. Transgend Health. 2019;4(1):209–216. doi: 10.1089/trgh.2019.0021 31552292 PMC6757240

[3] Mansh M, White W, Gee-Tong L, et al. Sexual and gender minority identity disclosure during undergraduate medical education: “in the closet” in medical school. Acad Med. 2015;90(5):634–644. doi: 10.1097/ACM.0000000000000657 25692563

[4] Meeks LM, Plegue M, Swenor BK, et al. The performance and trajectory of medical students with disabilities: results from a multisite, Multicohort Study. Acad Med. 2022;97(3):389. doi: 10.1097/ACM.0000000000004510 34817411 PMC8855952

[5] Wilby KJ, De Chun L, Ye R, et al. Students’ experiences with racism during the COVID-19 pandemic. Acad Med. 2021;96(1):e4–e5. doi: 10.1097/ACM.0000000000003801 33031112 PMC7565251

[6] Wyatt TR, Rockich-Winston N, Taylor TR, et al. What does context have to do with anything? A study of professional identity formation in physician-trainees considered underrepresented in medicine. Acad Med. 2020;95(10):1587–1593. doi: 10.1097/ACM.0000000000003192 32079956

[7] Blalock AE, Smith MC, Patterson BR, et al. “I might not fit that doctor image”: Ideal worker norms and women medical students. Med Educ. 2022;56(3):339–348. doi: 10.1111/medu.14709 34862660

[8] Johnson M, Seide W, Green-Dixon A, et al. Black students’ perception of belonging: A focus group approach with black students at the uniformed services university of the health sciences. *Int J Med Students*. 2021;9(2):124–128. doi: 10.5195/ijms.2021.877

[9] Kern DE A six-step approach to curriculum development. *Curriculum development for medical education: A six-step approach*. Published online 2016:5–9.

[10] Halman M, Baker L, Ng S. Using critical consciousness to inform health professions education: A literature review. Perspect Med Educ. 2017;6(1):12–20. doi: 10.1007/S40037-016-0324-Y 28050879 PMC5285284

[11] Elo S, Kyngäs H. The qualitative content analysis process. J Adv Nurs. 2008;62(1):107–115. doi: 10.1111/j.1365-2648.2007.04569.x 18352969

[12] Ho CP, Chong A, Narayan A, et al. Mitigating Asian American bias and xenophobia in response to the coronavirus pandemic: how you can be an upstander. J Am Coll Radiol. 2020;17(12):1692–1694. doi: 10.1016/j.jacr.2020.09.030 33035504 PMC7538066

[13] Woods FA, Ruscher JB. ‘Calling-out’ vs. ‘calling-in’ prejudice: confrontation style affects inferred motive and expected outcomes. Br J Soc Psychol. 2021;60(1):50–73. doi: 10.1111/bjso.12405 32633003

[14] Anderson RK. Burned out or burned through? the costs of student affairs diversity work. J Stud Aff Res Pract. 2021;58(4):359–371. doi: 10.1080/19496591.2020.1822853

[15] Pemberton A, Kisamore J. Assessing burnout in diversity and inclusion professionals. Equal Divers Incl: An Inter J. 2023;42(1):38–52. doi: 10.1108/EDI-12-2020-0360

