# Supplementary material for: Making allyship visible: evaluation of a faculty development DEI curriculum
Source: Med Educ Online. 2023 Jul 30;28(1):2241182. doi: 10.1080/10872981.2023.2241182 (PMC10392238; doi:10.1080/10872981.2023.2241182)
Supplement: Supplementary file 1 [file ZMEO_A_2241182_SM7335.odt]

Supplementary Material 1

Allyship Procedures

The procedures below were created to aid new facilitators not familiar with the Faculty Development infrastructure for tracking and the overall organization of the FAC.  We have a Faculty Development Self-Service Site (FDSSS) where faculty learners can sign-up for sessions, facilitators confirm attendance, and enduring materials are uploaded. 

- Scheduling Workshops

  - Schedule 2 workshops for the first week of each month (same workshop x 2) - try to do at least 6 months at a time at least 30 days prior to the next planned workshop

  - Send schedule to all involved facilitators and administrators- consider also sending a google invite at time of scheduling as a place holder

  - Enter workshops into the FDSSS

- Running Workshops

  - Send Zoom links for workshops to the facilitators for each workshop

  - Prepare for workshop (to include any breakout discussions) and ensure Poll Everywhere is functional if you will be using it

  - Ensure participants keep camera on

  - Close the registration and email a pdf of the class roster the day prior to the event

  - Take attendance

  - At the end of the workshop, remind attendees of the time and date of the related journal club

- Scheduling Journal Club

  - Schedule journal clubs out at least 6 months

  - Reach out to Allies or members of the diversity committee to solicit journal club presenters

  - At the end of the first week of each month, advertise the journal club via email groups and the Allyship journal club distribution list with the date, topic, article, and media. Include dates of future journal clubs and login information in this email.

  - Within 7 days of the journal club, send an email reminder. Include dates of future journal clubs and login information in this email.

- Running Journal Club

  - Update Allyship Journal Club slide deck for your workshop. Ensure that any new Allies are updated and congratulated.

  - On the last slide, ensure that there is a reminder to submit journal club reflections 

- Processing Completion

  - After attendees submit journal club reflections and final reflections, log it into the Allyship Reflections spreadsheet and check workshop credit hours

  - If an individual has completed the Allyship Curriculum, complete a digital Allyship certificate and print a hardcopy of the Allyship certificate 

  - Send a congratulations email to the Ally 

  - Present certificates and Allyship pins to Allies.

Supplemental Materials 2 

Program Evaluation Questionnaire

- What was your overall opinion of the activity? [Excellent 5, Good 4, Average 3, Fair 2, Poor 1]

- The material presented in the faculty development session was

- Please provide comments/suggestions for the class you attended.

- What did you learn about [title of workshop] today?

- Please rate the quality of the following presenters and provide comments.[ Excellent 5, Good 4, Average 3, Fair 2, Poor 1]

- I will use the knowledge and skills presented in the faculty development session in my job [Never, Occasionally, Sometimes, Often, Daily]

- Any comments for USU regarding faculty issues globally and faculty developments?

Supplemental Materials 3 

Workshop Evaluation Questionnaire

- Which courses did you take within the Allyship program? 

  - Addressing racism in medicine

  - Cross-cultural mentorship

  - Recognizing and responding to microaggressions 

  - Language of diversity

  - Step up: commit to health care equity

  - Fortify your learning climate

  - Operationalizing solutions to implicit bias 

  - Establishing trust in the teacher-learner relationship

  - Building better: equity in recruiting and selecting future colleagues

  - It’s no small thing: the challenges of small-group teaching

- To what extent has the Allyship program impacted your teaching practice? 

  - To a great extent

  - Somewhat

  - Very little

  - Not at all

- Which of the following specific changes have you made? As a result of participating in the Allyship program, I have… (Check all that apply)

  - Developed training materials based on the Allyship program for my teaching and practice context(s).

  - Started using language from the Allyship program in my daily practice (e.g., minority tax, microaggressions, implicit bias).

  - Educated colleagues on issues or topics related to the Allyship program, either formally or informally.

  - Discussed issues or topics from the Allyship program with URiM learners.

  - Had meetings regarding issues or topics from the Allyship program with non-URiM learners.

  - Added topics from the Allyship program to my research program.

  - Developed evaluation or QI measures based on what I learned in the Allyship program.

  - Considered the minority tax when assigning responsibilities.

  - Practiced cross-cultural mentorship.

  - Used reflection tools and models to teach social determinants of health.

  - Spoken with colleagues or community members about ways to address healthcare disparities.

  - Responded to a microaggression in real time.

  - Implemented measures to address systemic biases in my context.

  - Taken steps to support women in medicine.

  - Implemented measures to develop a culture of trust in my context.

  - Other

- As a result of the Allyship program, how confident do you feel in your ability to teach learners who are underrepresented in medicine?

  - Extremely confident

  - Very confident

  - Moderately confident

  - Slightly confident

  - Not confident at all

- As a result of the Allyship program, how confident do you feel in your ability to mentor learners who are different from you?

  - Extremely confident

  - Very confident

  - Moderately confident

  - Slightly confident

  - Not confident at all

- As a result of the Allyship program, how confident do you feel in your ability to teach about topics from the Allyship program? 

  - Extremely confident

  - Very confident

  - Moderately confident

  - Slightly confident

  - Not confident at all

- Please share briefly one thing you are most proud of that you have done since you began the allyship program. 

- What has been the most helpful aspect of the program for your teaching practice? 

- How would you recommend we improve the program?

- Is there anything else you would like to share with us about the program?
